# Crystal structure of the diglycidyl ether of eugenol

**DOI:** 10.1107/S2056989017005370

**Published:** 2017-04-13

**Authors:** Jordan Vigier, Camille François, Sylvie Pourchet, Gilles Boni, Laurent Plasseraud, Vincent Placet, Stéphane Fontaine, Hélène Cattey

**Affiliations:** a ICMUB CNRS UMR 6302, Université de Bourgogne Franche-Comté, Faculté des Sciences, 9 avenue Alain Savary, 21000 Dijon, France; b FEMTO Institute, Applied Mechanics Department, UMR CNRS 6174, Université de Bourgogne Franche-Comté, 24 Chemin de l’Epitaphe, 25000 Besançon, France; c DRIVE Laboratory, Institut Supérieur de l’Automobile et des Transports, 49 Rue Melle Bourgeois, 58027 Nevers, France

**Keywords:** crystal structure, oxirane, bio-based mol­ecule, eugenol derivative, ep­oxy thermoset prepolymer, hydrogen bonding

## Abstract

The diep­oxy monomer, C_13_H_16_O_4_ {**DGE-Eu**; systematic name: 2-[3-meth­oxy-4-(oxiran-2-ylmeth­oxy)benz­yl]oxirane}, was synthesized from eugenol by a three-step reaction. It consists of a 1,2,4-tris­ubstituted benzene ring substituted by diglycidyl ether, a meth­oxy group and a methyl­oxirane group. The three-membered oxirane rings are inclined to the benzene ring by 61.0 (3) and 27.9 (3)°. The methyl­ene C atom of one of the two terminal epoxide rings is positionally disordered [refined occupancy ratio = 0.69 (1):0.31 (1)]. In the crystal, mol­ecules are linked by C—H⋯O hydrogen bonds, forming layers parallel to the *ab* plane. The layers are linked by C—H⋯π inter­actions, forming a three-dimensional network.

## Abstract 2

The diep­oxy monomer (**DGE-Eu**) was synthesized from eugenol by a three-step reaction. It consists of a 1,2,4-tris­ubstituted benzene ring substituted by diglycidyl ether, a meth­oxy group and a methyl­oxirane group. The three-membered oxirane rings are inclined to the benzene ring by 61.0 (3) and 27.9 (3)°. In the crystal, mol­ecules are linked by C—H⋯O hydrogen bonds, forming layers parallel to the *ab* plane.

## Chemical context   

The past two decades have witnessed an increasing inter­est in the environmental quest for the replacement of petroleum-based chemicals by monomers from renewable resources. Advances in particular in the catalytic conversion of biomass have led to a wide range of useful platform mol­ecules (Besson *et al.*, 2014 ▸). This sustainable approach is also strongly considered in the field of polymer synthesis (Gandini *et al.*, 2016 ▸). In the specific domain of ep­oxy thermosets, numerous studies have been conducted in order to find alternatives to the diglycidyl ether of bis­phenol A (BADGE), which is the main building-block used for formulation resins (Auvergne *et al.*, 2014 ▸). Classically, the synthetic approach is based on the functionalization of bio-sourced mol­ecules by the grafting of glycidyl ether groups. In this context and in our ongoing studies on the chemical modification of bio-based building blocks for material applications (Mhanna *et al.*, 2014 ▸; Bigot *et al.*, 2016 ▸; François *et al.*, 2016 ▸), we report herein on the synthesis and crystal structure of the diglycidyl ether of eugenol (**DGE-Eu**), prepared from eugenol in a three-step synthesis (Qin *et al.*, 2014 ▸).
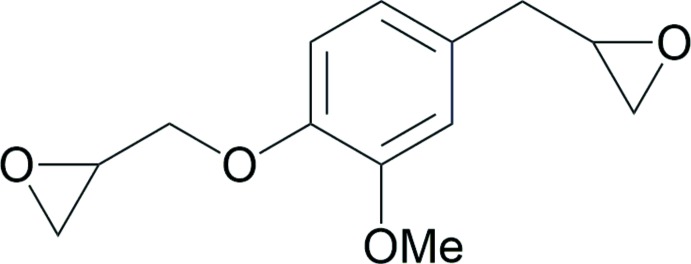


## Structural commentary   

**Figure 1:**
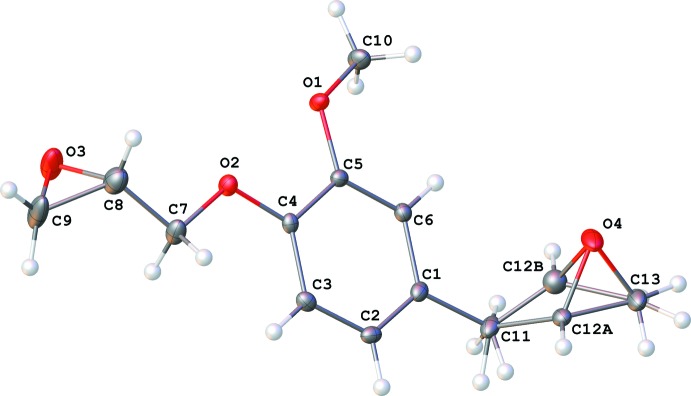
A view of the mol­ecular structure of the title compound (**DGE-Eu**), with the atom labelling. Displacement ellipsoids are drawn at the 50% probability level. The major and minor components of atom C12 (C12*A*/C12*B*) are shown.

The title compound (**DGE-EU**), has an asymmetrical structure, which is depicted in Fig. 1 ▸. It is composed of a benzene ring with three oxygenated functional groups: (i) 2-[(λ^1^-oxidan­yl)meth­yl]oxirane, (ii) meth­oxy and (iii) 2-methyl­oxirane. While atoms O1, O2 and C11 lie in the plane of the benzene ring, the meth­oxy group (O1/C5/C10) is inclined to the benzene ring by 11.2 (3)°. The two oxirane rings (O3/C8/C9 and O4/C12*A*/C13) are inclined to the benzene ring by 61.0 (3) and 27.9 (3)°, respectively. The mol­ecule shows disorder with an occupation factor equal to 0.69 (1) for the major component of the methyl­ene group (C12*A*) of the oxirane ring (C11/C12/O3). Such disorder is commonly observed for diglycidyl ether derivatives (CSD; Groom *et al.*, 2016 ▸).

## Supra­molecular features   

**Figure 2:**
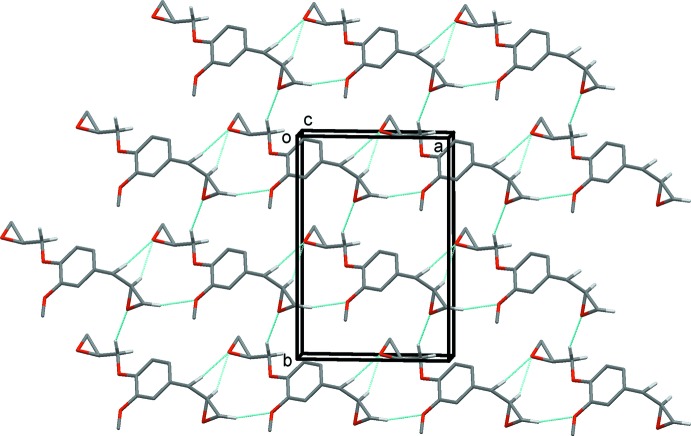
Crystal packing of **DGE-Eu** viewed along the *c* axis, showing the layer-like C—H⋯O hydrogen-bonded network (dashed lines; see Table 1 ▸). Only the major component of atom C12 (C12*A*) is shown. For clarity, only H atoms H7*B*, H11*C*, H12*A*, H13*B* and H7*A* have been included.

**Figure 3:**
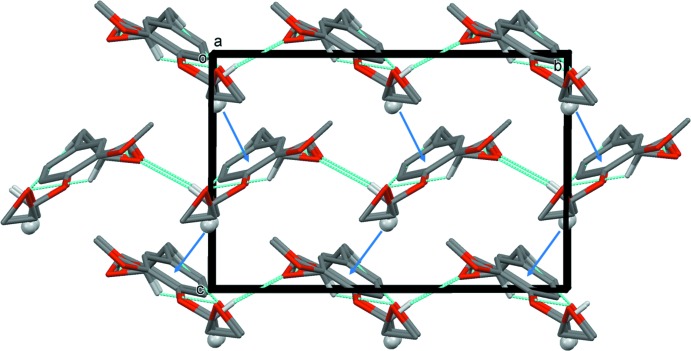
Crystal packing of **DGE-Eu**, viewed along the *a* axis, showing the layer-like C—H⋯O hydrogen-bonded networks linked by C—H⋯π inter­actions (dashed lines and blue arrows, respectively; see Table 1 ▸). For clarity, only H atoms H7*B*, H11*C*, H12*A*, H13*B* and H7*A* (grey ball) have been included. Only the major component of atom C12 (C12*A*) is shown.

The crystal packing of **DGE-Eu** viewed along the *c*-axis is depicted in Fig. 2 ▸. All oxygen atoms of **DGE-Eu** are involved in C—H⋯O hydrogen bonds with surrounding mol­ecules, forming layers lying parallel to the *ab* plane (Fig. 2 ▸ and Table 1 ▸). In addition, the layers are linked C—H⋯π inter­actions, with the C7–H7*A* group positioned almost orthogonally to the benzene ring, so forming a three-dimensional network (Table 1 ▸ and Fig. 3 ▸).

## Database survey   

To date, and to the best of our knowledge, only nine crystallographic structures comprising diglycidyl ether-substituted benzene ring moieties have been deposited in the Cambridge Structural Database (WebCSD v1.1.2, update 2017-04-05; Groom *et al.*, 2016 ▸). They include 2,2-bis­(3,5-di­bromo-4-hy­droxy­benzene)­propane diglycidyl ether (COMNEX: Saf’yanov *et al.*, 1984 ▸), 2,2-bis­[4-(oxiran-2-ylmeth­oxy)-3,5-di­bromo­phen­yl]propane (COMNEY: Cheban *et al.*, 1985 ▸), diglycidyl ether of bis­phenol A (DGEBPA: Flippen-Anderson & Gilardi, 1980 ▸; DGEBPA01: Heinemann *et al.*, 1993 ▸; DGEBPA10: Flippen-Anderson & Gilardi, 1981 ▸), *p*-di(2,3-ep­oxy­prop­yloxy)benzene (EOXHQE: Saf’yanov *et al.*, 1977 ▸), 2,2′-[1,3-phenyl­ene-bis­(oxymethyl­ene)]bis­(oxirane) (FITWOU: Bocelli & Grenier-Loustalot, 1987 ▸), 2-(4-{4-[4-(oxiran-2-ylmeth­oxy)phen­oxy]phen­yl}phen­oxy­meth­yl)oxir­ane (LAQTII: Song *et al.*, 2012 ▸) and 10-[2,5-bis­(2,3-ep­oxy-1-prop­oxy)phen­yl]-9-oxa-10-phosphaphenanthren-10-one (LIPSOS: Cho *et al.*, 1999 ▸). In some of these compounds, an ep­oxy ring is disordered, which is also observed for the title compound **DGE-Eu**. In terms of application, these compounds are used as precursors of thermosetting resins. The polymerization process involving the ep­oxy rings occurs in the presence of amines and acid anhydrides and leads to cross-linked rigid materials.

## Synthesis and crystallization   

**Figure 4:**
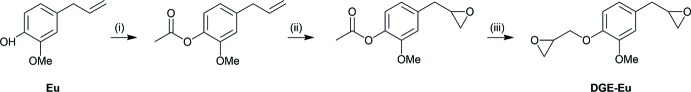
Reagents and conditions for the synthesis of **DGE-Eu** from eugenol (Qin *et al.*, 2014 ▸): (i) acetic anhydride, 358 K; (ii) *m*-chloro­per­oxy­benzoic acid, CH_2_Cl_2_, room temperature; (iii) epi­chloro­hydrin, NaOH, C_2_H_5_OH, 358 K.

The title compound was prepared from a commercial source of eugenol (Sigma–Aldrich), according to a three-step procedure previously reported in the literature (Qin *et al.*, 2014 ▸). The details of the synthesis of the title compound are summarized in Fig. 4 ▸. Following purification by silica gel column chromatography, colourless prismatic crystals were obtained by slow evaporation of an ethyl acetate solution, and were finally characterized as **DGE-Eu**.

## Refinement details   

Crystal data, data collection and structure refinement details are summarized in Table 2 ▸. The H atoms were placed at calculated positions and refined using a riding model: C—H = 0.95–1.00 Å with *U*_iso_(H) = 1.5*U*_eq_(C-meth­yl) and 1.2*U*_eq_(C) for other H atoms. Atom C12 atom of the ep­oxy­propane (oxirane) group (C11/C12/O3) was found to be disordered over two positions with a refined occupancy ratio of 0.69 (1): 0.31 (1) for atoms C12*A*:C12*B*.

## Supplementary Material

Crystal structure: contains datablock(s) global, I. DOI: 10.1107/S2056989017005370/su5360sup1.cif

Structure factors: contains datablock(s) I. DOI: 10.1107/S2056989017005370/su5360Isup2.hkl

Supporting information file. DOI: 10.1107/S2056989017005370/su5360Isup3.cml

CCDC reference: 1543288

Additional supporting information:  crystallographic information; 3D view; checkCIF report

## Figures and Tables

**Table 1:** Hydrogen-bond geometry (Å, °) *Cg* is the centroid of the benzene ring (C1–C6).

*D*—H⋯*A*	*D*—H	H⋯*A*	*D*⋯*A*	*D*—H⋯*A*
C7—H7*B*⋯O4^i^	0.99	2.53	3.452 (4)	155
C11—H11*C*⋯O3^ii^	0.99	2.43	3.413 (4)	170
C13—H13*B*⋯O1^ii^	0.99	2.57	3.358 (4)	136
C12*A*—H12*A*⋯O3^ii^	1.00	2.45	3.177 (5)	129
C7—H7*A*⋯*Cg* ^iii^	0.99	2.57	3.465 (4)	150

Symmetry codes: (i) 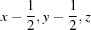; (ii) 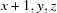; (iii) 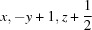.

**Table 2:** Experimental details

Crystal data	
Chemical formula	C_13_H_16_O_4_
*M*_r_	236.26
Crystal system, space group	Monoclinic, *C* *c*
Temperature (K)	115
*a*, *b*, *c* (Å)	9.8262 (5), 13.4434 (7), 9.4251 (8)
β (°)	109.897 (2)
*V* (Å^3^)	1170.71 (13)
*Z*	4
Radiation type	Mo *K*α
μ (mm^−1^)	0.10
Crystal size (mm)	0.4 × 0.35 × 0.3

Data collection	
Diffractometer	Bruker APEXII CCD
Absorption correction	Multi-scan (*SADABS*; Bruker, 2014 ▸)
*T*_min_, *T*_max_	0.700, 0.747
No. of measured, independent and observed [*I* > 2σ(*I*)] reflections	18897, 2680, 2586
*R*_int_	0.021
(sin θ/λ)_max_ (Å^−1^)	0.650

Refinement	
*R*[*F* ^2^ > 2σ(*F* ^2^)], *wR*(*F* ^2^), *S*	0.044, 0.121, 1.07
No. of reflections	2680
No. of parameters	160
No. of restraints	2
H-atom treatment	H-atom parameters constrained
Δρ_max_, Δρ_min_ (e Å^−3^)	0.69, −0.28
Absolute structure	Flack *x* determined using 1234 quotients [(*I* ^+^)−(*I* ^−^)]/[(*I* ^+^)+(*I* ^−^)] (Parsons *et al.*, 2013 ▸)
Absolute structure parameter	0.20 (18)

Computer programs: *APEX2* and *SAINT* (Bruker, 2014 ▸), *SHELXT2015* (Sheldrick, 2015*a* ▸), *SHELXL2015* (Sheldrick, 2015*b* ▸), *OLEX2* (Dolomanov *et al.*, 2009 ▸) and *Mercury* (Macrae *et al.*, 2008 ▸).

## supplementary crystallographic information

### Crystal data

**Table:**

C_13_H_16_O_4_	*F*(000) = 504
*M**_r_* = 236.26	*D*_x_ = 1.340 Mg m^−^^3^
Monoclinic, *C**c*	Mo *K*α radiation, λ = 0.71073 Å
Hall symbol: C -2yc	Cell parameters from 9992 reflections
*a* = 9.8262 (5) Å	θ = 2.7–33.0°
*b* = 13.4434 (7) Å	µ = 0.10 mm^−^^1^
*c* = 9.4251 (8) Å	*T* = 115 K
β = 109.897 (2)°	Prism, colourless
*V* = 1170.71 (13) Å^3^	0.4 × 0.35 × 0.3 mm
*Z* = 4	

### Data collection

**Table:**

Bruker APEXII CCD diffractometer	2680 independent reflections
Radiation source: X-ray tube, Siemens KFF Mo 2K-180	2586 reflections with *I* > 2σ(*I*)
Graphite monochromator	*R*_int_ = 0.021
φ and ω scans	θ_max_ = 27.5°, θ_min_ = 2.7°
Absorption correction: multi-scan (SADABS; Bruker, 2014)	*h* = −12→12
*T*_min_ = 0.700, *T*_max_ = 0.747	*k* = −17→17
18897 measured reflections	*l* = −12→12

### Refinement

**Table:**

Refinement on *F*^2^	Secondary atom site location: difference Fourier map
Least-squares matrix: full	Hydrogen site location: inferred from neighbouring sites
*R*[*F*^2^ > 2σ(*F*^2^)] = 0.044	H-atom parameters constrained
*wR*(*F*^2^) = 0.121	*w* = 1/[σ^2^(*F*_o_^2^) + (0.0641*P*)^2^ + 1.3035*P*] where *P* = (*F*_o_^2^ + 2*F*_c_^2^)/3
*S* = 1.07	(Δ/σ)_max_ < 0.001
2680 reflections	Δρ_max_ = 0.69 e Å^−^^3^
160 parameters	Δρ_min_ = −0.28 e Å^−^^3^
2 restraints	Absolute structure: Flack *x* determined using 1234 quotients [(*I*^+^)-(*I*^-^)]/[(*I*^+^)+(*I*^-^)] (Parsons *et al.*, 2013)
Primary atom site location: structure-invariant direct methods	Absolute structure parameter: 0.20 (18)

### Special details

**Table:**

Geometry. All esds (except the esd in the dihedral angle between two l.s. planes) are estimated using the full covariance matrix. The cell esds are taken into account individually in the estimation of esds in distances, angles and torsion angles; correlations between esds in cell parameters are only used when they are defined by crystal symmetry. An approximate (isotropic) treatment of cell esds is used for estimating esds involving l.s. planes.

### Fractional atomic coordinates and isotropic or equivalent isotropic displacement parameters (Å^2^)

**Table:**

	*x*	*y*	*z*	*U*_iso_*/*U*_eq_	Occ. (<1)
O1	0.3186 (2)	0.75130 (14)	0.4046 (2)	0.0215 (4)	
O2	0.3043 (2)	0.59282 (15)	0.5548 (2)	0.0221 (4)	
O3	0.0296 (3)	0.4884 (2)	0.5717 (4)	0.0511 (9)	
O4	0.8644 (3)	0.79876 (18)	0.4484 (3)	0.0406 (7)	
C1	0.6472 (3)	0.6219 (2)	0.3926 (3)	0.0204 (6)	
C2	0.6394 (3)	0.5387 (2)	0.4744 (3)	0.0203 (5)	
H2	0.7121	0.4890	0.4925	0.024*	
C3	0.5262 (3)	0.5260 (2)	0.5313 (3)	0.0204 (6)	
H3	0.5220	0.4677	0.5867	0.025*	
C4	0.4203 (3)	0.5984 (2)	0.5068 (3)	0.0169 (5)	
C5	0.4272 (3)	0.6842 (2)	0.4242 (3)	0.0171 (5)	
C6	0.5397 (3)	0.6949 (2)	0.3680 (3)	0.0187 (5)	
H6	0.5441	0.7527	0.3119	0.022*	
C7	0.2935 (3)	0.5033 (3)	0.6333 (4)	0.0287 (7)	
H7A	0.3828	0.4935	0.7213	0.034*	
H7B	0.2807	0.4452	0.5655	0.034*	
C8	0.1678 (4)	0.5132 (3)	0.6838 (5)	0.0382 (8)	
H8	0.1691	0.5701	0.7526	0.046*	
C9	0.0890 (4)	0.4237 (4)	0.6998 (5)	0.0522 (12)	
H9A	0.1274	0.3584	0.6826	0.063*	
H9B	0.0428	0.4238	0.7782	0.063*	
C10	0.3084 (3)	0.8287 (2)	0.2985 (4)	0.0269 (6)	
H10A	0.3053	0.7997	0.2020	0.040*	
H10B	0.3929	0.8724	0.3366	0.040*	
H10C	0.2201	0.8674	0.2839	0.040*	
C11	0.7701 (3)	0.6367 (3)	0.3324 (4)	0.0278 (7)	
H11A	0.8071	0.5708	0.3159	0.033*	0.690 (11)
H11B	0.7323	0.6706	0.2334	0.033*	0.690 (11)
H11C	0.8536	0.5974	0.3965	0.033*	0.310 (11)
H11D	0.7401	0.6074	0.2299	0.033*	0.310 (11)
C13	0.9712 (4)	0.7691 (2)	0.3826 (4)	0.0324 (7)	
H13A	0.9471	0.7781	0.2725	0.039*	0.690 (11)
H13B	1.0745	0.7788	0.4434	0.039*	0.690 (11)
H13C	1.0477	0.7222	0.4401	0.039*	0.310 (11)
H13D	1.0014	0.8187	0.3216	0.039*	0.310 (11)
C12A	0.8911 (4)	0.6954 (3)	0.4331 (5)	0.0203 (11)	0.690 (11)
H12A	0.9476	0.6614	0.5295	0.024*	0.690 (11)
C12B	0.8184 (12)	0.7317 (8)	0.3230 (12)	0.032 (3)*	0.310 (11)
H12B	0.7600	0.7658	0.2274	0.038*	0.310 (11)

### Atomic displacement parameters (Å^2^)

**Table:**

	*U*^11^	*U*^22^	*U*^33^	*U*^12^	*U*^13^	*U*^23^
O1	0.0200 (9)	0.0204 (9)	0.0264 (10)	0.0043 (7)	0.0107 (7)	0.0072 (8)
O2	0.0216 (10)	0.0238 (10)	0.0222 (9)	−0.0004 (8)	0.0089 (8)	0.0056 (8)
O3	0.0229 (12)	0.0500 (17)	0.070 (2)	−0.0009 (11)	0.0028 (12)	0.0306 (16)
O4	0.0437 (14)	0.0246 (12)	0.0679 (18)	−0.0074 (10)	0.0379 (13)	−0.0174 (12)
C1	0.0174 (12)	0.0230 (13)	0.0210 (13)	−0.0033 (11)	0.0067 (10)	−0.0078 (11)
C2	0.0186 (12)	0.0202 (12)	0.0194 (12)	0.0029 (10)	0.0029 (10)	−0.0036 (10)
C3	0.0226 (13)	0.0187 (12)	0.0168 (12)	−0.0001 (10)	0.0026 (10)	0.0014 (9)
C4	0.0165 (12)	0.0195 (13)	0.0129 (11)	−0.0036 (9)	0.0026 (10)	0.0003 (9)
C5	0.0160 (11)	0.0170 (12)	0.0167 (11)	0.0005 (9)	0.0035 (9)	−0.0010 (9)
C6	0.0191 (12)	0.0175 (12)	0.0198 (12)	−0.0019 (9)	0.0068 (10)	−0.0008 (9)
C7	0.0218 (14)	0.0326 (16)	0.0299 (15)	0.0008 (12)	0.0067 (11)	0.0146 (13)
C8	0.0330 (17)	0.042 (2)	0.0429 (19)	0.0016 (15)	0.0170 (15)	0.0098 (16)
C9	0.0247 (16)	0.056 (2)	0.075 (3)	0.0001 (16)	0.0159 (18)	0.040 (2)
C10	0.0292 (15)	0.0234 (14)	0.0304 (15)	0.0051 (12)	0.0133 (12)	0.0108 (12)
C11	0.0215 (13)	0.0314 (16)	0.0346 (16)	−0.0047 (12)	0.0146 (12)	−0.0140 (12)
C13	0.0323 (16)	0.0259 (14)	0.049 (2)	−0.0044 (13)	0.0267 (15)	−0.0069 (14)
C12A	0.021 (2)	0.020 (2)	0.023 (2)	0.0008 (15)	0.0118 (16)	−0.0013 (15)

### Geometric parameters (Å, º)

**Table:**

O1—C5	1.361 (3)	C8—H8	1.0000
O1—C10	1.423 (3)	C8—C9	1.467 (5)
O2—C4	1.364 (3)	C9—H9A	0.9900
O2—C7	1.435 (3)	C9—H9B	0.9900
O3—C8	1.447 (5)	C10—H10A	0.9800
O3—C9	1.441 (4)	C10—H10B	0.9800
O4—C13	1.444 (4)	C10—H10C	0.9800
O4—C12A	1.430 (4)	C11—H11A	0.9900
O4—C12B	1.432 (11)	C11—H11B	0.9900
C1—C2	1.374 (4)	C11—H11C	0.9900
C1—C6	1.402 (4)	C11—H11D	0.9900
C1—C11	1.513 (4)	C11—C12A	1.473 (5)
C2—H2	0.9500	C11—C12B	1.375 (12)
C2—C3	1.400 (4)	C13—H13A	0.9900
C3—H3	0.9500	C13—H13B	0.9900
C3—C4	1.386 (4)	C13—H13C	0.9900
C4—C5	1.406 (4)	C13—H13D	0.9900
C5—C6	1.386 (4)	C13—C12A	1.443 (5)
C6—H6	0.9500	C13—C12B	1.499 (12)
C7—H7A	0.9900	C12A—H12A	1.0000
C7—H7B	0.9900	C12B—H12B	1.0000
C7—C8	1.473 (5)		
			
C5—O1—C10	116.4 (2)	O1—C10—H10B	109.5
C4—O2—C7	115.7 (2)	O1—C10—H10C	109.5
C9—O3—C8	61.1 (2)	H10A—C10—H10B	109.5
C12A—O4—C13	60.3 (2)	H10A—C10—H10C	109.5
C12B—O4—C13	62.8 (5)	H10B—C10—H10C	109.5
C2—C1—C6	118.7 (3)	C1—C11—H11A	108.8
C2—C1—C11	121.4 (3)	C1—C11—H11B	108.8
C6—C1—C11	119.9 (3)	C1—C11—H11C	107.6
C1—C2—H2	119.4	C1—C11—H11D	107.6
C1—C2—C3	121.1 (3)	H11A—C11—H11B	107.7
C3—C2—H2	119.4	H11C—C11—H11D	107.0
C2—C3—H3	120.0	C12A—C11—C1	113.6 (3)
C4—C3—C2	120.0 (2)	C12A—C11—H11A	108.8
C4—C3—H3	120.0	C12A—C11—H11B	108.8
O2—C4—C3	124.8 (2)	C12B—C11—C1	118.9 (5)
O2—C4—C5	115.6 (2)	C12B—C11—H11C	107.6
C3—C4—C5	119.6 (2)	C12B—C11—H11D	107.6
O1—C5—C4	115.8 (2)	O4—C13—H13A	117.8
O1—C5—C6	124.7 (2)	O4—C13—H13B	117.8
C6—C5—C4	119.5 (2)	O4—C13—H13C	118.0
C1—C6—H6	119.4	O4—C13—H13D	118.0
C5—C6—C1	121.2 (2)	O4—C13—C12B	58.2 (5)
C5—C6—H6	119.4	H13A—C13—H13B	115.0
O2—C7—H7A	110.1	H13C—C13—H13D	115.1
O2—C7—H7B	110.1	C12A—C13—O4	59.4 (2)
O2—C7—C8	108.0 (3)	C12A—C13—H13A	117.8
H7A—C7—H7B	108.4	C12A—C13—H13B	117.8
C8—C7—H7A	110.1	C12B—C13—H13C	118.0
C8—C7—H7B	110.1	C12B—C13—H13D	118.0
O3—C8—C7	115.2 (3)	O4—C12A—C11	116.8 (3)
O3—C8—H8	116.8	O4—C12A—C13	60.4 (2)
O3—C8—C9	59.3 (2)	O4—C12A—H12A	114.7
C7—C8—H8	116.8	C11—C12A—H12A	114.7
C9—C8—C7	119.3 (4)	C13—C12A—C11	124.5 (4)
C9—C8—H8	116.8	C13—C12A—H12A	114.7
O3—C9—C8	59.7 (2)	O4—C12B—C13	59.0 (5)
O3—C9—H9A	117.8	O4—C12B—H12B	112.2
O3—C9—H9B	117.8	C11—C12B—O4	123.4 (8)
C8—C9—H9A	117.8	C11—C12B—C13	127.6 (8)
C8—C9—H9B	117.8	C11—C12B—H12B	112.2
H9A—C9—H9B	114.9	C13—C12B—H12B	112.2
O1—C10—H10A	109.5		
			
O1—C5—C6—C1	−179.6 (3)	C3—C4—C5—O1	179.6 (2)
O2—C4—C5—O1	0.9 (3)	C3—C4—C5—C6	0.2 (4)
O2—C4—C5—C6	−178.5 (2)	C4—O2—C7—C8	176.7 (3)
O2—C7—C8—O3	83.5 (4)	C4—C5—C6—C1	−0.2 (4)
O2—C7—C8—C9	150.9 (4)	C6—C1—C2—C3	0.6 (4)
O4—C13—C12A—C11	103.8 (4)	C6—C1—C11—C12A	84.8 (4)
O4—C13—C12B—C11	−110.4 (10)	C6—C1—C11—C12B	31.8 (7)
C1—C2—C3—C4	−0.7 (4)	C7—O2—C4—C3	−1.3 (4)
C1—C11—C12A—O4	−69.5 (4)	C7—O2—C4—C5	177.3 (2)
C1—C11—C12A—C13	−140.5 (3)	C7—C8—C9—O3	−103.5 (4)
C1—C11—C12B—O4	54.5 (11)	C9—O3—C8—C7	110.4 (4)
C1—C11—C12B—C13	128.8 (8)	C10—O1—C5—C4	−168.6 (2)
C2—C1—C6—C5	−0.1 (4)	C10—O1—C5—C6	10.8 (4)
C2—C1—C11—C12A	−93.8 (4)	C11—C1—C2—C3	179.3 (2)
C2—C1—C11—C12B	−146.8 (6)	C11—C1—C6—C5	−178.8 (2)
C2—C3—C4—O2	178.8 (2)	C13—O4—C12A—C11	−116.3 (4)
C2—C3—C4—C5	0.3 (4)	C13—O4—C12B—C11	117.2 (10)

### Hydrogen-bond geometry (Å, º)

Cg is the centroid of the benzene ring (C1–C6).

**Table:**

*D*—H···*A*	*D*—H	H···*A*	*D*···*A*	*D*—H···*A*
C7—H7*B*···O4^i^	0.99	2.53	3.452 (4)	155
C11—H11*C*···O3^ii^	0.99	2.43	3.413 (4)	170
C13—H13*B*···O1^ii^	0.99	2.57	3.358 (4)	136
C12*A*—H12*A*···O3^ii^	1.00	2.45	3.177 (5)	129
C7—H7*A*···*Cg*^iii^	0.99	2.57	3.465 (4)	150

Symmetry codes: (i) *x*−1/2, *y*−1/2, *z*; (ii) *x*+1, *y*, *z*; (iii) *x*, −*y*+1, *z*+1/2.
